# S53. COMPARISON OF RALOXIFENE AND ISRADIPINE AS AN ADJUNCTIVE TREATMENT IN COGNITIVE DEFICITS OF PATIENTS WITH SCHIZOPHRENIA

**DOI:** 10.1093/schbul/sby018.840

**Published:** 2018-04-01

**Authors:** Bita Vahdani, Alireza Armani kian, Abdolreza Esmaeilzadeh, Saeedeh Zenoozian, Vida Yousefi, Saeedeh Mazloomzadeh

**Affiliations:** Zanjan University of Medical Sciences

## Abstract

**Background:**

Cognitive impairment is the most important feature of schizophrenia that leads to severe social and functional disability. Improving neurocognitive physiopathologic aspect of schizophrenia is a current challenge to identify the pathway to develop goal directed clinical interventions in practice. In the current study we investigated the effect of raloxifine as a selective estrogen modulator and isradipine as a voltage gated L type calcium channel blocker on the enhancement of schizophrenic patients’ cognitive deficits.

**Methods:**

We designed a double blind randomized, parallel, placebo controlled clinical trials. 60 patients with schizophrenia randomized in 3 specific groups. The first group received isradipine 5 mg, the second raloxifine 60 mg and the third placebo for 6 consequent weeks, in the same shape capsules, 2 times a day, alongside treatment with the conventional antipsychotics. The initial and final lab tests, ECG, as well as cognitive tests in specific domains such as attention, processing speed, executive function and verbal memory were carried out.

**Results:**

Our findings, revealed a remarkable association between adjunctive treatment of raloxifine in verbal memory deficits. moreover, isradipine treatment indicated significant improvement relative to placebo in verbal memory as well as attention dysfunction in some variables of the Stroop test. However, no effect was observed in processing speed and executive function deficits.

**Discussion:**

The study provides the first evidence to our knowledge, which isradipine as a novel therapy was associated with improvement in verbal memory and attention, both related to hippocampal and cerebellar activity. Overall, further investigation is necessary to determine the various ways of the both drugs performance in the brain.

